# Do lifestyle factors influence risk of breast cancer recurrence in Korean women?: a cross-sectional survey

**DOI:** 10.4069/kjwhn.2022.06.08

**Published:** 2022-06-29

**Authors:** So-Jung Park, Hye-Ah Yeom

**Affiliations:** 1 Department of Nursing, The Catholic University of Korea, Seoul St. Mary’s Hospital, Seoul, Korea; 2 College of Nursing, The Catholic University of Korea, Seoul, Korea

**Keywords:** Breast, Life style, Neoplasms, Recurrence, Risk factors

## Abstract

**Purpose:**

This study aimed to investigate the influencing factors of breast cancer recurrence by comparing the risk factors and lifestyle patterns related to breast cancer in Korean women with and without recurrence.

**Methods:**

This cross-sectional survey comprised 241 Korean women diagnosed with breast cancer who had received follow-up treatment. Participants were recruited from a university hospital in Seoul and an online social media platform for breast cancer patients. Data were collected either via online or a paper survey, using a structured questionnaire that included general and disease-related characteristics and lifestyle behaviors. Data were analyzed using descriptive statistics, univariate analysis, and logistic regression.

**Results:**

Recurrence of breast cancer was influenced by four factors; childbirth experience, consumption of green/yellow vegetables, drinking behavior, and recovery from fatigue after sleep.Prevalence of recurrent breast cancer was associated with no childbirth experience (OR=2.29, *p*=.010), fewer green/yellow vegetables (OR=0.71, *p*=.008), drinking behavior (OR=0.24, *p*=.001), and a lower level of recovery from fatigue after sleep (OR=0.51, *p*<.001).

**Conclusion:**

Aside from having experienced childbirth, this study identified several modifiable factors that influence breast cancer recurrence. Increasing green/yellow vegetable intake, alleviating fatigue, and reducing alcohol intake are important. Intervention strategies in clinical research and practice can be applied to address risk factors and reduce the prevalence of recurrent breast cancer.

## Introduction

According to the National Cancer Registry in South Korea (hereafter Korea), the life expectancy of the general population in Korea is approximately 83 years, with an estimated 34.2% lifetime incidence of cancer in the female population [1]. Breast cancer morbidity in Korea, in particular, has steadily increased due to late childbirth, decreased breastfeeding, increased alcohol consumption, westernized eating habits, and obesity [1]. The number of breast cancer cases in Korea comprises approximately 100 in men and 23,547 in women each year, and breast cancer is the most common cancer among Korean women [1].

The 5-year relative survival of patients with breast cancer in Korea increased from 79.2% between 1993 and 1995 to 93.3% between 2014 and 2018 [1]. However, even after completion of treatment for breast cancer, patients may still experience a variety of secondary physical problems, including metastatic cancer, lymphedema, peripheral neuropathy, and cardiovascular diseases [2]. Also, patients continue to experience anxiety regarding recurrence or metastasis even after 5 years of posttreatment survival and tend to perceive recurrence in relation to death [2]. Anxiety and uncertainty concerning the future due to potential recurrence is the most significant factor leading to psychological distress in patients with breast cancer [3]; therefore, there is a need to explore methods to maximize the prevention of recurrence and promote quality of life throughout patients’ lives following cancer treatment [4].

Determining causative factors of breast cancer remains unclear, but a variety of factors, such as personal characteristics and lifestyle patterns, have been reported in previous studies. These risk factors include family history, estrogen exposure, old age, absence of childbirth and breastfeeding experience, radiation exposure, alcohol consumption, a high-fat diet, obesity, and exposure to endocrine disruptors [5,6].

The factors related to breast cancer recurrence, however, are far less understood than the risk factors for early breast cancer. It is known that lifestyle affects not only the incidence but also the likelihood of breast cancer recurrence [7]. The identified factors related to breast cancer incidence that may influence recurrence include age [8-12], family history [9-10,13,14], disease history [8,9,10], education level [9], family income [9,15], occupation [9,13,16], menstruation, childbirth and breastfeeding experience [8,9,11-13], eating habits [7,17-22], obesity [7,8,10,11,22-24], alcohol consumption [8,10,11,19,22,24], smoking [2,9,11,14,19,22], physical activity and rest [7,9,12,19,22,25], stress management [17,26], hair dyeing [9], brassiere wearing time [27], breast plastic surgery [27], and the duration of having taken oral contraceptive pills or menopausal hormonetherapy [8,11,13,26].

In Korea, while studies have investigated the risk factors and lifestyle related to early breast cancer incidence, studies regarding the influencing factors of breast cancer recurrence are lacking. In addition to treatment-related factors, identifying lifestyle factors correlated with recurrent breast cancer is crucial to designing nursing interventions for patients. For long-term survivors of cancer, despite the importance of a healthy daily lifestyle to prevent recurrence or metastasis, they tend to seek advice from a wide array of sources of varying quality [28]. It is essential that they are provided with consistent and reliable information regarding measures to minimize cancer recurrence. Thus, the purpose of this study was to investigate the influencing factors of breast cancer recurrence by comparing the risk factors and lifestyle patterns related to breast cancer in women with and without recurrence and to provide basic data for developing interventions to help prevent breast cancer recurrence.

## Methods

Ethics statement: This study was approved by the Institutional Review Board of The Catholic University of Korea (KC20QASI0878). Obtaining written informed consent was exempted because the survey was completely anonymous and considering the online survey design.

### Study design

This cross-sectional survey employed a descriptive correlational design to investigate the influencing factors of breast cancer recurrence by comparing the risk factors and lifestyle patterns related to breast cancer in women with and without recurrence. This report followed the STROBE (Strengthening the Reporting of Observational Studies in Epideiology) reporting guidelines (https://www.strobe-statement.org/).

### Participants

The participants in this study comprised 241 adult women with a confirmed diagnosis of breast cancer receiving treatment or in posttreatment follow-up monitoring. The patients were either members of an online community of patients with breast cancer or those admitted to or visiting The Catholic University of Korea, Seoul St. Mary’s Hospital in Seoul, Korea. The women with breast cancer recurrence, the recurrent group, comprised 114 patients whose mid-treatment or posttreatment follow-up test results showed recurrence or metastasis in the breast on the side where breast cancer had been originally detected, or the contralateral breast, or in an entirely different organ. The nonrecurrent group comprised 127 patients. The inclusion criteria were women aged 19 years or above, who were capable of reading and responding to online or written questionnaires. The G*Power 3.1.9 program was used to calculate the appropriate number of participants required, based on power (1-β) of .80, significance level of .05, and an effect size of .15. Because there are sparse reports on the influence of lifestyle factors on recurrence of breast cancer, a conventional low level was set for the effect size [17]. A minimum of 104 participants in each group were required [29], meeting the requirement for sample size for this study.

### Instrument

The variables in this study were measured using a structured questionnaire that was modified and revised by the investigator based on the influencing factors of breast cancer reported in previous studies [26]. Due to the absence of the instrument solely focused on the influencing factors for recurrent breast cancer, we included individual variables that were found to be correlated with the incidence of primary breast cancer in the literature. Two types of influencing factors were measured; general characteristics and lifestyle factors. The face validity of the measurements was assured by expert panel review by two nursing professionals including one nursing professor and one clinical nurse with expertise in the breast cancer unit.

Questions on influencing factors related to general characteristics consisted of nine questions in total, including three questions on estrogen exposure (menstruation, childbirth, and breastfeeding) and six questions on age, family history, comorbid diseases before the diagnosis of breast cancer, education level, household income, and occupation. To derive these nine variables included in this study, a literature search was conducted using keywords such as ‘breast cancer risk factor,’ ‘breast cancer lifestyle,’ ‘breast cancer recurrence,’ ‘breast neoplasm risk factor,’ ‘breast neoplasm lifestyle,’ and ‘breast neoplasm recurrence.’ General characteristics that were reported as significantly associated with breast cancer recurrence in at least one previous study were included as the variables of this study.

Data on influencing factors related to lifestyle were obtained through 37 questions in relation to four conceptual domains: eating habits (21 items), physical activity (five items), stress management (three items), and other factors (eight items) which include overweight, alcohol consumption, smoking, hair dyeing, hours of brassiere wearing, plastic surgery on breast, the duration of having taken menopausal hormone therapy, and the duration of having taken oral contraceptive pill. The questions concerning eating habits, physical activity, and stress management were rated on a 5-point Likert scale (1, strongly agree to 5, strongly disagree). The questions on overeating, unbalanced eating, and eating instant foods were reverse coded (1, strongly disagree to 5, strongly agree). Higher scores (possible range, 29–145) indicated more engagement in healthy lifestyles in the domain of eating habits, physical activity, and stress management. Obesity was measured in terms of body mass index (BMI), obtained by dividing weight (kg) by the square of height (m^2^) (underweight, <18.5; normal weight, 18.5–24.99; overweight, ≥25.0) [30]. Participants in the recurrent group were instructed to record their lifestyle for the period of initial diagnosis to the time before diagnosis of recurrence.

### Data collection

The data were collected between December 15, 2020 and February 19, 2021. Recruitment advertisement was done by posting a link to a breast cancer support community using a Google questionnaire developed by the researchers so that potential participants could visit the link to voluntarily participate in the anonymized online survey. Patients with breast cancer admitted to or visiting the hospital were also invited to the study, after the consent of the nursing department at The Catholic University of Korea Seoul St. Mary’s Hospital regarding data collection. Individuals who were contacted in-person and verbally consented to participate were provided a paper questionnaire, which took 10 to 15 minutes. Participants were asked to recall their general lifestyles after initial diagnosis of breast cancer.

### Data analysis

The collected data were analyzed using IBM SPSS for Windows ver. 25.0 (IBM Corp., Armonk, NY, USA). The personal characteristics of the participants are presented in terms of frequency, percentage, mean, and standard deviation. Variations in risk factors and lifestyle were examined using a chi-square test between the recurrent and nonrecurrent groups. In terms of breast cancer recurrence factors, variables showing a significant difference in the univariate analysis were used as independent variables in the logistic regression analysis. The questionnaires were designed to be anonymous, and the data are managed in compliance with the Personal Information Protection Act (Korea) [31].

## Results

### General and clinical characteristics of participants

The mean age of the participants was 49.45 years, of whom the majority were married (78.0%) and were college or university graduates (63.5%). Less than one-third were currently working (28.2%) and the mean monthly income was 4.308 million Korean won (KRW; approximately 3,340 US dollars), which is slightly greater than the mean average household income for 2020 [32]. Stage 2 breast cancer was the most frequent form of cancer (n=109, 45.2%). In the recurrent group (n=114), 91 participants (37.8%) reported one recurrence, 17 participants (7.1%) reported two recurrences, and 6 (2.5%) reported three or more recurrences (Table 1).

The general characteristics were compared between the recurrent and nonrecurrent (n=127) groups, and the results showed significant differences in terms of a history of benign breast cancer, childbirth experience, and breastfeeding experience. The percentage of participants with a history of benign breast cancer was higher in the recurrent group (25.4%) than in the nonrecurrent group (12.6%) (*p*=.012). The percentage of participants without childbirth experience was higher in the recurrent group (39.5%) than in the nonrecurrent group (21.3%) (*p*=.002), and the percentage of participants without breastfeeding experience was higher in the recurrent group (53.5%) than in the nonrecurrent group (37.8%) (*p*=.014) (Table 2).

### Comparison of lifestyle between women with and without recurrent breast cancer

The analysis of eating habits showed that, compared to the nonrecurrent group, the recurrent group had irregular meals (*p*=.001), skipped breakfast more often (*p*=.008), and tended to overeat (*p*=.041). The recurrent group also had an imbalanced diet (*p*=.007), ate fewer green and yellow vegetables (*p*=.003), and consumed instant foods to a higher degree (*p*=.046) (Table 3).

Regular physical activity was practiced less in the recurrent group (*p*=.005). The recurrent group was also found to take insufficient rest when recovering from fatigue (*p*=.003) and reported experiencing an incomplete recovery after sleep (*p*<.001). In terms of alcohol consumption and smoking, the rate of those who were engaged in these behaviors was higher in the recurrent group (*p*=.001). In addition, the recurrent group reported a longer time wearing a brassiere all day (*p*=.017). Concerning stress management, no significant differences were observed between the two groups (Table 4).

### Factors influencing breast cancer recurrence

Fifteen factors identified as showing significant differences in the univariate analysis were used as independent variables in the logistic regression analysis. These factors included experience of childbirth, breastfeeding, a history of benign breast cancer, regular meals, eating breakfast, overeating, imbalanced diet, eating green and yellow vegetables, eating instant foods, regular exercise, recovery from fatigue through sufficient rest, recovery from fatigue after sleep, alcohol consumption, smoking, and brassiere wearing. The logistic regression analysis showed 70.3% accuracy, while the Hosmer and Lemeshow test showed *p*=.574, verifying the fit of the regression model.

The regression analysis also showed that the influencing factors influencing the recurrence of breast cancer were childbirth (Odds ratio [OR]=2.29, *p*=.010), eating green and yellow vegetables (OR=0.71, *p*=.008), alcohol consumption (OR=0.24, *p*=.001), and degree of recovery from fatigue after sleep (OR=0.51, *p*<.001). Specifically, the risk of breast cancer recurrence increased by 2.29 times in participants without childbirth experience and decreased by 0.71 times with the consumption of green and yellow vegetables. Recurrence decreased by 0.24 times in participants who did not consume alcohol, and by 0.51 times in participants who reported complete recovery from fatigue after sleep (Table 5).

## Discussion

This study aimed to identify factors influencing breast cancer recurrence by comparing the personal characteristics and lifestyle patterns associated with breast cancer between women with and without recurrent breast cancer. The results showed that the factors influencing the recurrence of breast cancer were experience of childbirth, eating green and yellow vegetables, alcohol consumption, and recovery from fatigue after sleep.

The recurrence of breast cancer increased by 2.285 times in women without childbirth experience, indicating that this factor affected breast cancer incidence as well as its recurrence. The experience or frequency of childbirth was reported in a previous study to be related to the incidence of initial diagnosis of breast cancer and miscarriage experiences [9]. In terms of recurrence of breast cancer, however, there is sparse literature on the correlations of recurrence of breast cancer with childbirth experiences. Therefore, the findings of this study should be further examined in future studies to confirm the influence of childbirth experience on the prevalence of recurrent breast cancer.

In this study, the recurrence of breast cancer decreased by 0.706 times as the consumption of green and yellow vegetables increased. Although direct comparison with the findings of previous research should be done with caution due to sparse literature on influencing factors of recurrent breast cancer, this result accords indirectly with previous studies reporting a low risk of prevalence of breast cancer and extended longevity in women with a more vegetable-centered diet [20,22]. It is also consistent with the fact that survival rates increased with reducing fat intake to 20% of energy and increasing consumption of fruits, vegetables, and grains [21]. Foods reported to reduce breast cancer risk are consistent with our results, e.g., green-yellow vegetables, fruits, and seaweed foods [20] and antioxidant vitamins A, C, E [8].

In this study, the risk of breast cancer recurrence decreased by 0.237 times in women who did not consume alcohol, which is in line with previous studies reporting an increase in the risk of recurrence after breast cancer diagnosis when alcohol is consumed [23]. In this study, while smoking was not found to be a significant factor, alcohol consumption and smoking were measured using a dichotomous variable. To identify specific associations of these variables with recurrent breast cancer, ample information on dose, type, duration of drinking and smoking behaviors needs to be obtained in future studies.

Regarding quality sleep, women who routinely sleep fewer hours may develop more breast cancer recurrence compared with women who sleep longer hours [25]. Given that the risk of breast cancer recurrence was found to decrease by 0.506 times with improved recovery from fatigue after sleep in this study, ensuring sufficient sleep and fatigue recovery is an important area of care for women with breast cancer.

The mean age of the participants in this study was 49.45 years, which reflects the Korean National Cancer Registry data that show the highest incidence of breast cancer occurs among women aged between 40 and 49 years [1]. In this study, age was not a factor influencing breast cancer recurrence, but in previous studies, the prognosis of breast cancer has been found to be relatively poor in younger adults [10], indicating a need for further studies on the relationship between age and breast cancer recurrence.

The risk of breast cancer recurrence has been reported to be greater in overweight or obese women [23] and as BMI increases [28]. In this study, however, BMI was not found to influence the recurrence of breast cancer, which accords with another study reporting a lack of correlation between weight gain and breast cancer recurrence [24]. In addition, although studies have reported a higher risk of breast cancer with a higher level of education [9], a higher level of household income [9,15], and having an occupation [9], these factors were not found to influence the recurrence of breast cancer in this study. Concerning occupation type, the risk of breast cancer has been reported to increase among female workers who perform night shifts [13]. As this study focused only on whether participants had an occupation rather than types of occupation, further studies are needed to assess the risk of breast cancer recurrence in relation to specific occupations.

This study had several limitations. First, it is possible that the level of understanding varied among individual respondents in relation to the use of online and offline questionnaires, which may have affected the results in the process of data collection. For example, the duration or time point for recall of lifestyle factors was not specified (last 6 months, etc.) in the questionnaire, which may have generated recall biases of the participants. Although the face validity of the questionnaire was obtained through expert panel review, caution is required as other types of validity were not possible. Second, cause-effect relationships could not be determined because the study used a cross-sectional descriptive design. Therefore, in the future, a longitudinal study with a larger sample size should be conducted to facilitate generalization of the results. Additionally, clinical variables such as cancer subtype information and the period after being diagnosed with cancer were not included because the data were collected based on self-reports from the patients, and exact information regarding clinical status was not available. Examining homogeneity in treatment status of breast cancer will be necessary to increase validity of the study findings in future studies. Thus, assessment of duration of maintaining lifestyle factors and clinical information through review of medical records will help to clarify factors influencing breast cancer recurrence. Because our sample size was relatively small, further research with a larger dataset will be beneficial. Overall, the influencing factors identified in this study for breast cancer recurrence should be considered as relevant intervention variables in follow-up studies to promote evidence-based practice for the prevention of breast cancer.

In conclusion, this study identified not having experienced childbirth, lack of eating green and yellow vegetables, alcohol consumption, and poor recovery from fatigue after sleep as factors influencing breast cancer recurrence. Noting that several are modifiable factors, healthcare professionals can use these findings to assess the lifestyle patterns of patients with breast cancer and implement appropriately targeted intervention plans to decrease the risk of breast cancer recurrence.

## Figures and Tables

**Table 1.** General characteristics of the participants (N=241)

Variable	Categories	Mean ± SD or n (%)
Education	≤Middle school	9 (3.7)
	High school	79 (32.8)
	≥College	153 (63.5)
Monthly income (×10^4^Korean won^†^)		427.42±284.72
Marital status	Married	188 (78.0)
	Single	44 (18.3)
	Divorced, bereaved	9 (3.7)
Recurrence of breast cancer	No	127 (52.7)
	1 time	91 (37.8)
	2 times	17 (7.1)
	3 times	6 (2.4)
Breast cancer stage at present time	0	3 (1.2)
	Ⅰ	51 (21.2)
	Ⅱ	109 (45.2)
	Ⅲ	45 (18.7)
	Ⅳ	33 (13.7)

† One million Korean won is approximately 800 US dollars.

**Table 2.** Comparison of general and clinical characteristics between women with and without recurrent breast cancer (N=241)

Characteristics	Categories	N (%)		
		Nonrecurrent (n=127)	Recurrent (n=114)	χ^2^(*p*)
Age (year)	<30	1 (0.8)	4 (3.5)	5.21 (.267)
	30–39	17 (13.4)	16 (14.0)	
	40–49	46 (36.2)	46 (40.4)	
	50–59	45 (35.4)	28 (24.6)	
	<60	18 (14.2)	20 (17.5)	
Family history of breast cancer	Yes	21 (16.5)	17 (14.9)	1.04 (.595)
	No	106 (83.5)	97 (85.1)	
Diagnosed disease before breast cancer	Benign breast disease	16 (12.6)	29 (25.4)	6.38 (.012)
	Others/none	111 (87.4)	85 (74.6)	
Education	≤Middle school	4 (3.1)	5 (4.4)	1.19 (.757)
	High school	41 (32.3)	38 (33.3)	
	≥College or above	82 (64.6)	71 (62.3)	
Occupation	Employed	38 (30.0)	42 (36.8)	1.30 (.255)
	Unemployed	89 (70.1)	72 (63.2)	
Menstruation status	Active	37 (29.1)	36 (31.6)	5.67 (.225)
	Non-active	90 (70.9)	78 (68.4)	
Childbirth experience	Yes	100 (78.7)	69 (60.5)	9.51 (.002)
	No	27 (21.3)	45 (39.5)	
Breastfeeding experience	Yes	79 (62.2)	53 (46.5)	5.99 (.014)
	No	48 (37.8)	61 (53.5)	

**Table 3.** Comparison of eating habits between women with and without recurrent breast cancer (N=241)

Eating habits prior to recurrence	Mean±SD		t (*p*)
	Nonrecurrent (n=127)	Recurrent (n=114)	
Regular meals	3.63±1.01	3.14±1.18	3.45 (.001)
Breakfast every day	3.57±1.29	3.10±1.42	2.68 (.008)
Often overeating	3.32±0.93	3.05±1.10	2.06 (.041)
Short meal time	3.18±1.03	2.94±1.18	1.63 (.104)
Imbalanced diet	3.50±1.03	3.11±1.18	2.72 (.007)
Eating a lot of salty food	3.33±0.88	3.11±0.92	1.86 (.065)
Eating a lot of meat	3.24±0.89	3.08±1.09	1.22 (.224)
Eating a lot of fresh fish	3.01±0.87	2.89±1.09	1.00 (.319)
Eating a lot of burnt food	3.82±0.83	3.61±1.00	1.80 (.239)
Eating a lot of fried food	3.32±1.07	3.20±1.06	0.88 (.382)
Eating a lot of milk products	3.50±0.92	3.34±0.90	1.37 (.171)
Eating a lot of beans	3.77±0.88	3.85±0.89	–0.62 (.534)
Eating a lot of green and yellow vegetables	3.42±0.86	3.07±0.96	2.96 (.003)
Eating a lot of fruits	3.47±0.91	3.40±0.91	0.58 (.561)
Eating a lot of seaweed-based foods	3.04±0.94	2.91±0.86	1.15 (.250)
Eating a lot of nuts	3.14±0.89	3.14±0.81	0.01 (.997)
Eating health supplements	3.04±1.29	2.77±1.19	1.71 (.089)
Eating a lot of instant foods	3.48±0.93	3.22±1.08	2.00 (.046)
Drinking coffee every day	2.79±1.34	2.68±1.33	0.64 (.520)
Drinking more than 1.5 L of water a day	3.22±1.06	2.97±1.08	1.76 (.081)
Eating a lot of hot (high temperature) food	2.90±0.98	2.92±0.91	–0.13 (.900)

**Table 4.** Comparison of lifestyle between women with and without recurrent breast cancer (N=241)

Domain	Variable	Categories	Mean±SD or n (%)		χ²/t (*p*)
			Nonrecurrent (n=127)	Recurrent (n=114)	
Physical activity and rest	Regular physical activity		3.29±1.10	2.88±1.11	2.83 (.005)
	Breathing exercise		2.80±1.12	2.53±1.11	1.85 (.065)
	Taking sufficient rest		3.59±0.90	3.22±1.01	2.95 (.003)
	Taking more than 8 hours of sleep		3.11±1.09	2.83±1.09	1.95 (.052)
	Fatigue recovery after sleep		3.37±0.91	2.79±1.03	4.56 (<.001)
Stress management	Optimistic personality		3.08±1.05	3.06±1.08	0.18 (.855)
	Being stressed out easily		2.59±1.00	2.53±1.09	0.47 (.639)
	Having relief from stress		2.99±0.93	2.82±0.92	1.39 (.165)
Other factors	Body mass index	Underweight	12 (9.5)	20 (17.5)	3.57(.168)
		Normal	93 (73.2)	74 (65.0)	
		Overweight	22 (17.3)	20 (17.5)	
	Alcohol drinking	Yes	10 (7.8)	28 (24.6)	11.87 (.001)
		No	117 (92.1)	86 (75.4)	
	Smoking	Yes	2 (1.6)	7 (6.1)	13.36 (.001)
		No	116 (91.3)	84 (73.7)	
		Quit	9 (7.1)	23 (20.2)	
	Hair dyeing	Periodic	29 (22.8)	31 (27.2)	4.04 (.133)
		Occasional	48 (37.8)	30 (26.3)	
		No	50 (39.4)	53 (46.5)	
	Hours of wearing a brassiere per day		9.91±8.00	12.35±7.74	–2.39 (.017)
	Receiving reconstructive surgery on breast	Yes	7 (5.5)	12 (10.5)	2.08 (.149)
		No	120 (94.5)	102 (89.5)	
	Menopausal hormone therapy	Yes	49 (38.6)	46 (40.4)	0.08 (.779)
		No	78 (61.4)	68 (59.6)	
	Months of having taken oral contraceptive pills		15.10±29.35	14.63±25.92	0.05 (.958)

**Table 5.** Factors influencing recurrence of breast cancer (N=241)

Variable (criteria)	B	SE	Wald	*p*	OR	95% CI
Childbirth, no^†^	0.83	0.32	6.70	.010	2.29	1.22–4.27
Eating green and yellow vegetable, strongly agree^†^	–0.35	0.20	3.07	.008	0.71	0.48–0.94
Alcohol drinking, no^†^	–1.44	0.42	11.79	.001	0.24	0.10–0.54
Fatigue recovery, strongly agree^†^	–0.68	0.18	14.29	<.001	0.51	0.36–0.72
Constant term	3.66	1.05	12.20	<.001	38.86	
Accuracy	0.073					
	Hosmer-Lemeshow; χ²=5.71, df=7, *p*=.574					

† References were childbirth (yes), green and yellow vegetables (strongly disagree), alcohol drinking (yes), and fatigue recovery (strongly disagree).

CI: Confidence interval; OR: odds ratio
